# Polysaccharide Composite Films Utilising Wood Waste

**DOI:** 10.3390/ma16176031

**Published:** 2023-09-02

**Authors:** Anita Kwaśniewska, Michał Świetlicki, Beata Kowalska, Grzegorz Gładyszewski

**Affiliations:** 1 Department of Applied Physics, Faculty of Mechanical Engineering, Lublin University of Technology, Nadbystrzycka 36, 20-618 Lublin, Poland; m.swietlicki@pollub.pl (M.Ś.); g.gladyszewski@pollub.pl (G.G.); 2 Department of Water Supply and Wastewater Disposal, Faculty of Environmental Engineering, Lublin University of Technology, Nadbystrzycka 40D, 20-618 Lublin, Poland; b.kowalska@pollub.pl

**Keywords:** biopolymer composite films, physical properties, wood dust, bio-based material

## Abstract

This study aimed to investigate the effect of raw waste pine wood dust (*Pinus sylvestris*) from furniture production on polysaccharide biopolymer film properties. The obtained biocomposite films produced via the casting method were prepared with 20% glycerol and 0%, 5%, 10%, 15%, 20%, and 25% of added wood dust in relation to the dry starch matter. Wood dust composition and particle size distribution analysis were performed. In order to evaluate the material surface properties, tests were carried out using an atomic force microscope (AFM) and a contact angle goniometer. Utilising uniaxial tensile test methodology, the values for both tensile strength and Young’s modulus were determined. In addition, the barrier properties, water solubility index, and colour were also investigated. The research showed that wood dust affected the functional parameters of the obtained biocomposites. A wood dust content increase causes the Young’s modulus value to rise with a progressive decrease in the max. strain. The filler did not change the films’ wetting properties, and each had a hydrophilic surface regardless of the additive amount. The bio-sourced composites obtained were non-toxic and environmentally neutral materials, suitable to be applied in the packaging industry as well as the agriculture sector.

## 1. Introduction

Over the past few years, environmental regulation has been significantly tightened, which has contributed to the implementation of innovative technologies to produce materials based on environmentally friendly components. The environmental impact of non-biodegradable plastics and the dwindling availability of fossil resources have catalysed a global pursuit of greener and more sustainable materials. Traditionally, the polymer sector has predominantly relied upon raw materials derived from petroleum sources or synthetically altered natural materials through chemical and thermal interventions. However, this approach is often environmentally inefficient and generates numerous by-products and environmentally hazardous waste.

Various research has been carried out into polysaccharide biopolymers, i.e., starch, pectin, alginate, and chitosan, to obtain nonpolluting green biodegradable materials [1,2,3,4]. These naturally abundant plant carbohydrate storage compounds present a highly suitable source material for synthesising thermoplastic polymers. This versatile material can be processed using standard thermoplastic processing methods, creating various products with varying shapes, sizes, and properties. It is well known that polysaccharide polymers are hygroscopic and have mechanical properties below those of synthetic polymers. To enhance their physical properties, a variety of functional additives, i.e., mineral, organic, synthetic, and natural, have been added to biopolymer matrices [5,6,7,8,9,10,11,12]. Biocomposites are the combination of the polymer that binds and protects the filler from external agents and the filler that modifies and enhances the matrix. Furthermore, the functional additive reduces the amount of raw material needed to make the proper material. Additionally, the efficiency of using natural resources increases by reducing production waste. This is in line with the circular economy trend that has been promoted recently. The availability of natural fibres and specific properties such as low relative density, renewability in nature, biodegradability, and low cost have widened the usage compared with other materials [13,14].

Accurate analyses were carried out on the wood particles’ impact on the physical properties of the synthetic polymers PP (polypropylene) [15,16,17], HDPE (high-density polyethylene) [18], PVA (polyvinyl alcohol) [19], and PVC (polyvinyl chloride) [20]. Nevertheless, due to their ecological advantages, natural fillers perfectly match natural polymers resulting in an eco-friendly and low-cost composite. For example, adding cellulose fibres to thermoplastic starch matrices increases mechanical properties and improves thermal resistance by enhancing the biocomposites’ glass transition temperature [21,22]. A similar effect was confirmed by Muller et al.: adding lignocellulosic fibres improved the stiffness and strength of the biocomposite [23]. A starch matrix filled with rubber wood dust also displayed better water resistance [24]. Neem wood sawdust addition increased composite stiffness and thermal degradation temperature while reducing water uptake with increasing filler content [25]. These composites offer improved mechanical, thermal, and barrier properties and can be used in various applications, improving the material economic viability and resource efficiency. Various types of waste are developed during wood processing into semi-finished and finished products. Industrial wood waste from coniferous or deciduous trees varies in type, form, and quality in the form of large and small pieces, shavings, sawdust, wood dust, or bark. At the same time, this waste can be an alternative source of valuable raw wood material. In Poland, the largest amount of industrial wood waste is generated in the sawmill industry—over 63% (edgings, sawdust, and bark). A total of 14% is accounted for by the furniture industry (wood dust, sawdust, and shavings), 13% comes from the wood-based panel industry (shavings, wood dust, and bark), and the pulp industry accounts for over 8% of the total amount of waste (mostly bark) [26].

In the realm of innovative polymeric materials, a recent course involves using unaltered source materials to obtain commodities characterised by minimal environmental impact. Within this framework, unmodified potato starch, as a widely available product, is a promising candidate for producing biodegradable polymers. For this matter, dust waste from furniture production, treated as an environmental problem, can be transformed into a valuable raw material without separation into individual fractions. The study assessed pine wood dust production waste as a functional biopolymer filler, free of any impurities or insignificant amounts of natural substances. The examined composite films were based on non-modified potato starch. In order to obtain thermoplastic starch (TPS), it was subjected to a high temperature in the presence of plasticisers, i.e., water and glycerol, providing disruption to its granular structure.

The surface topography, wettability, and mechanical and barrier properties were investigated via atomic force microscopy (AFM), contact angle, micro-tensile tests, vapour permeability, and the water solubility index.

## 2. Materials and Methods

### 2.1. Materials

The biopolymer films were prepared from native potato starch produced by Melvit S.A. (Warsaw, Poland). The starch was a raw product unmodified in any chemical, thermophysical, or enzymatic treatments. The solvent in which the polymer solution was prepared was distilled water. To become thermoplastic, native starch needs the presence of plasticisers. In this work, Glycerol 99.5%, produced by Avant Performance, was used.

Wood dust was obtained in the production process of wooden furniture from pine wood (*Pinus sylvestris*). The first quality class was that without knots and resin sockets and with a moisture content of 6–8% (for use in residential premises). The sawn pine timber was mechanically processed by cutting friezes and slats for furniture production. It was thereafter passed through a four-sided planer to level and obtain the required dimensions (thickness and width).

After cutting to length, the finished elements were sent to a wide belt sander DMC SD 30 (Scm Group, Rimini, Italy) equipped with two sanding belts of different grits (the first belt with a grit of P80—grain 201 µm and the second belt with a grit of P120—grain 125 µm). The technological process of grinding elements was carried out to smooth the surface before painting. Grinding allowances are usually from 0.5 mm to 2 mm on the element. Sawdust is generated on the first and second grinding rollers during the grinding process. Dust is extracted from the machine and separated in special extraction filters. The sawdust for testing was taken from the middle part of such a filter. Therefore, it was clean and free from varnish dust or shavings generated in this furniture factory in other mechanical processing departments.

#### 2.1.1. The Preparation of the Biopolymer Films

The starch-based films were made via the casting method [27]. A total of 120 mL of distilled water was used to prepare an aqueous solution of starch and plasticiser. Wood dust was added to the attained biopolymer solution, and to make it uniform, it was treated with an ultrasonic homogeniser for 180 s at 25 °C. The obtained suspension was treated with a magnetic stirrer rotating at 150 rpm and heated up to 80 °C for 30 min. Afterwards, the composite solution was poured into moulds and kept in a climatic chamber until the solvent evaporated. Drying was carried out at 23 °C at 50% RH for 4 days. The films were conditioned in a desiccator. Samples were prepared with 20% glycerol and 0%, 5%, 10%, 15%, 20%, and 25% of added wood dust in relation to the dry starch matter. Examined samples were marked as W0–W25.

#### 2.1.2. The Wood Dust Properties/Parameters

The wood dust was examined in terms of its fibre components: neutral detergent fibre (NDF), acid detergent fibre (ADF), and acid detergent lignin (ADL). NDF, ADF, and ADL were determined in milled samples via the Van Soest method [28] using an ANKOM 2000 Fiber Analyzer (ANKOM Technology, Macedon, NY, USA) for extraction of the fibre components. The content of hemicellulose and cellulose in the dust was calculated in the following way: cellulose = ADF − ADL, hemicellulose = NDF − ADF. Chemical analyses of the wood dust were carried out in three replications.

The size and quantity of wood dust particles were evaluated using the Morphologi^®^ G3/G3-ID particle characterisation system (Malvern Instruments Ltd., Worcestershire, UK). An 11 cm^3^ sample was dispersed onto a glass plate using the integral Sample Dispersion Unit (SDU) at a pressure of 0.5 bar. The correct parameters of dispersion pressure, injection time, and settling time ensured the powder’s uniform dispersion. Two magnifications, 48× and 240×, for particle imaging were used to ensure that any larger particles or agglomerates were captured. Particle size distribution measurements were taken from 5 repetitions.

### 2.2. Surface Properties/Morphology

#### 2.2.1. Atomic Force Microscopy

The films were scanned using an atomic force microscope (AFM, MuliMode 8, Bruker Corporation, Santa Barbara, CA, USA) with a ScanAsyst-Air probe (Bruker Corporation, Santa Barbara, CA, USA) in the PeakForce Tapping mode. Three noncontinuous regions of 10 μm by 10 μm from each sample were scanned with a scanning rate of 1 Hz. The surface roughness Sa and root mean square (RMS) roughness Sq were obtained after performing the 1st-order flattening operation (Nanoscope Analysis, Bruker Corporation, Santa Barbara, CA, USA) data to remove any tilt.

#### 2.2.2. Colour Measurement

The films’ colour was determined using an X-Rite PANTONE spectrocolorimeter (X-Rite Inc., Grand Rapids, MI, USA) and expressed on the CIE L*a*b scale, an international standard for colour measurements. Within the CIELAB colour scale, the parameters of luminosity (L*: degree of lightness) and chromaticity (a*: red–green and b*: yellow–blue) were measured. A calibration plate with known L* 97.133, a* −0.699, and b* 1.918 parameters was used to measure the background. The degree of colour saturation C (Chroma) and the hue H (Hue) were also calculated based on the values of a* and b*.

#### 2.2.3. Surface Wettability

The static sessile drop method was used to characterise the wettability of the surface films using an Attension Theta Lite optical goniometer (Biolin Scientific, Espoo, Finland). The contact angle was determined based on the geometry of the water drop on the tested surface. A 6 μL water drop was placed on each film using a chromatography fixed-needle syringe, type 3, and Gauge 22s. For each time on a new surface, the image was captured 3 s after deposition. The measurement series consisted of 5 drops and was conducted at 23 °C and 50% RH. The mean value read from both sides of the drop was taken as the measured value.

### 2.3. Barrier Properties and Solubility Index

The gravimetric method was used to determine the water vapour permeability coefficient (WVP), which is based on measuring the mass loss from a film-covered vessel over time under strictly defined conditions. The containers were filled with distilled water and sealed with Parafilm^®^M film. The surface area (A) of the film was equal to 9.616 × 104 m^2^. The examined samples were weighed and kept in a climate chamber at 23 °C and 40% relative humidity. The test was conducted for 7 days, with daily weight measurements. The WVP was calculated using Equation (1) [29]:

WVP = (Δm·e)/(Δt·A·Δp),(1)

where Δm/Δt is the weight of moisture loss per unit of time determined from the slope obtained from the regression analysis of weight loss data versus time; A is the film area exposed to the vapour; e is the film thickness; and Δp is the water vapour pressure difference between the two sides of the film.

The thicknesses of the films were determined using an electronic micrometre. Data from 20 random positions per film were obtained, and average values were used in calculations.

The water solubility index (WSI) is defined as the percentage of the dry matter of the film, which is solubilised in water after 24 h and calculated as follows:

WVP = ((m_1_ − m_2_)/m_1_)·100%, (2)

where m_1_ is the initial dry film weight, and m_2_ is the final dry film weight.

To determine the dry mass, the biocomposite films were cut into 20 mm × 20 mm pieces and subsequently dried in an oven at 50 °C for 24 h. Next, samples were placed into a beaker filled with 50 mL of H_2_O_dest_ at 24 °C and stirred at 50 rpm for 24 h. Then, the insoluble films were dried again and weighed. For each film, measurements were conducted in triplicate.

### 2.4. Mechanical Properties

The measurements were conducted using the Deben Microtest (Deben Ltd. Suffolk, UK) with a 200 N maximum load. In addition, Young’s modulus, tensile strength, and maximum strain were measured using the uniaxial tensile test. Tested samples were rectangular, with a width of 3.4 mm and a length of 20 mm. The initial distance between the grips was 10.5 mm, and films from each formulation were assayed five times at a constant speed equal to 0.5 mm/min.

### 2.5. Statistical Analyses

Statistical analyses, including a histogram and a boxplot of the data obtained from the measurements, were performed using the software package Statistica 13.1 (TIBCO Software Inc., Palo Alto, CA, USA).

## 3. Results and Discussion

**Figure 1:**
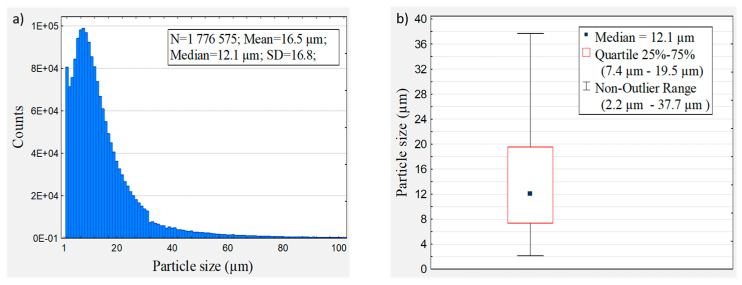
Wood dust histogram of particle size distribution (**a**) and boxplot of particle size distribution (**b**), where lower and upper quartiles appear as a box, min. and max. non-outlier values appear as whiskers, and outlier values are not shown. The size and quantity of wood dust particles were evaluated using the Morphologi^®^ G3/G3-ID particle characterisation system.

The particle size characterises particulate materials, whereas the particle size distribution represents the relative proportions of different grain sizes. In this regard, wood dust was examined via microscopy image analysis and the obtained results are given in Figure 1. Common wood dust consists of large particles of more than 10 μm in size. However, smaller particles may occur after sanding wood with fine sandpaper.

The examined wood dust was obtained after using P120 sanded paper. It was found that the median particle size of pine dust was 12.1 μm, and 95% of the counts’ particles consisted within the range of 2.2 μm to 37.7 μm. The results are shown in the histogram, and a representative boxplot of the particle size distributions is given. The boxplot does not present outlier values that represent 5% of wood dust particles.

The composition of pine wood dust was determined, and the percentages of substance content are shown in Table 1. The examined wood dust consists of a higher ratio of hemicellulose H and cellulose C (~55%) to lignin L (~31%). Elmas et al. reported similar values of fibre components for *Pinus sylvestris* for cellulose (50.2%) and lignin (28.3%), respectively [30]. It is well known that microbial degradation H and C are faster than lignin [31]. Therefore, it can be assumed that the susceptibility of the biocomposite to microbial decomposition would also increase when increasing the amount of the additive. The average dry matter value in the tested samples was 95.93%.

**Figure 2:**
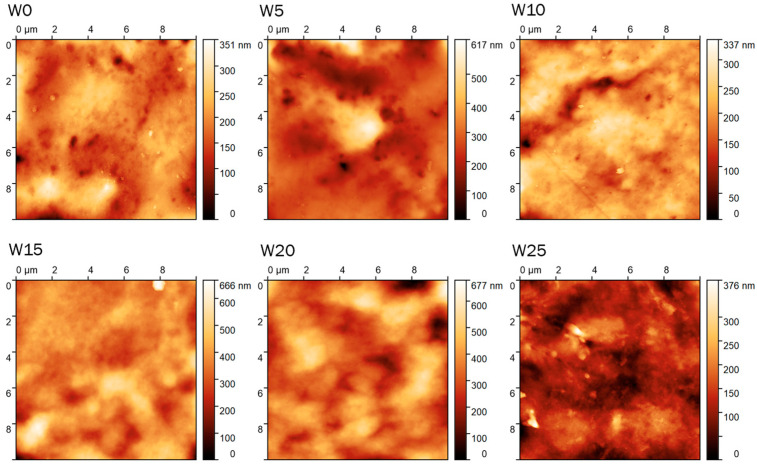
AFM scans (10 µm × 10 µm, height) of the films with wood dust content.

The research presented shows that the average roughness Sa and the root mean square roughness Sq of the examined composites decreased in the presence of sawdust (Figure 2). Interestingly, adding only 5% resulted in a notable reduction in both roughness parameters, wherein a comparably spectacular (large) effect was not observed for a higher amount of the additive.

Our research revealed that in the presence of wood dust, the surface observed on the nanoscale is more uniform and flat, irrespective of the additive content. Due to the relatively small scan area, it is difficult to explain the observed changes clearly, but the AFM scans indicate the existence of, at least locally, homogeneous and evenly distributed material. To the best of our knowledge, the roughness of such composites was not assessed on the nanoscale; however, the work of S. Kumar et al. demonstrated that the composites of polylactic acid (PLA) and polyvinyl chloride (PVC), which were reinforced with wood dust, showed lower roughness in the presence of 5% wood dust compared to the roughness observed for the 10% additive [32].

Due to the small scan area compared to the filler particle size, the roughness measurements referred more to changes in the polymer matrix itself, excluding the particle filler. Adding wood dust into the biopolymer caused the binding of polymer chains, which may reflect a decrease in the surface roughness value of biocomposite films. The AFM scans indicated the existence of, at least locally, homogeneous and evenly distributed material.

The water contact angle was used to evaluate the surface wettability of each film. As shown in Table 2, the addition of wood dust altered the measured values of the contact angle for all biocomposite films. However, both the base and the biocomposite films were characterised by contact angle values of less than 90 degrees. Thus, the addition of wood dust did not change the wetting properties of the tested films, and all of them were characterised by high water wettability, i.e., hydrophilicity.

In order to determine the effect of wood dust on the colour of the obtained biocomposite films, a colorimeter measurement was carried out. The results are shown in Table 3. The most lightness was measured for W0, and with the additive increase, the value of the parameter L decreased until reaching the value of 89.958. Parameters a* and b* also showed variability for examined samples, and biocomposite films tend to have yellowish tones with an increasing amount of wood dust.

Solubility determines the number of soluble polysaccharides realised from the biopolymer matrix. As all tested films were prepared the same, the amount of degraded, more-soluble starch portions was equal in all cases. Therefore, changes in the solubility of biocomposite films result mainly from additional adhesive interactions (interfacial adhesion) between the wood dust and the biopolymer matrix. As Dányádi et al. reported, interfacial adhesion depends on the surface energy, and for pine wood, due to its high cellulose content, it is low and ranges from 40 to 210 mJ/m^2^ [15].

**Figure 3:**
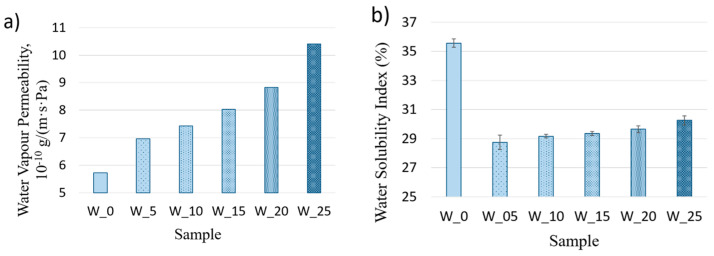
Water vapour permeability (**a**) and water solubility index (**b**); data given are mean ± SD.

However, wood dust particles can form an insoluble barrier for the starch matrix. Therefore, fortifying the biopolymer matrix with the addition of pine wood dust reduced the solubility of the obtained biocomposites. Figure 3 shows that the increase in the amount of additive decreases the films’ vapour barrier properties.

An opposite correlation was obtained by examining the films’ water vapour permeability. In this case, an increase in the additive resulted in increases in WPI from 5.72 to 10.4 × 10^−10^ g/(m·s·Pa). Since water vapour transfers through the hydrophilic part of the matrix, the film permeability depends on the amount of –OH groups. As the wood dust addition did not cause structural changes in the starch, the number of OH bonds was equal for all of the films. Therefore, the increase in vapour permeability for composite films arises from macro-phase separation formed between the wood dust particles themselves. Similar results were reported by El Halal et al. for cellulose-incorporated starch films. In this study, the increase in the value of cellulose addition caused a decrease in the value of the water vapour permeability. A similar trend was observed for the water solubility index, where the increase in the additive corresponded to a reduction in its value [33].

Interfacial interactions between the fillers and the matrix are reflected in the composite films’ mechanical properties. In order to determine the mechanical parameters, a uniaxial tensile test was conducted. Strain versus tensile force were recorded afterwards, and the tensile strength σmax, max. strain εmax, and elastic modulus E were calculated. Since the stress–strain curve of all samples did not contain a distinctly identifiable transition point between the elastic and plastic deformation, Young’s modulus was ascertained using the tangent angle of the initial curve segment’s slope. The progression of the stress–strain curve persisted until it reached the maximum stress at break [34]. The determined parameters and their standard deviation (SD) are given in Table 4. As can be observed, an increase in the amount of wood dust causes a decrease in the value ε_max_ for tested biocomposites. The film without the additive reached a max. strain of 0.42, while for W25, this value decreased to 0.19. Muller et al. also observed a similar decrease in the strain value for TPS composites with wood particles, where composites with 10% wood particles have a value five-fold lower [23]. A decrease in deformability due to wood dust reinforcement causes an increase in Young’s modulus. For composite films, it increased almost twice compared to the base film. The modulus values did not differ significantly for W5-W15 and were in the range of 42–46 MPa, respectively. However, the optimal content of the addition to improve the E parameter (52.36 MPa) was 20%. Further increases in the wood dust amount resulted in a decrease in Young’s modulus and reached the smallest value, 38 MPa, for W25. The tensile strength of biocomposite films was not affected significantly by the addition of wood dust, and the values oscillated in the range from 2.56 MPa to 2.78 MPa. A total of 20% of the filler content increased the strength of the base starch matrix by 17% and reached the maximum measured value. A further increase in the wood dust content decreased the tensile strength. Interfacial interactions between the dust itself probably cause such properties. Its increased content decreased the number of adhesive bonds between the wood dust and the biopolymer matrix, decreasing the stress transfer ability. The increased water vapour permeability confirmed this for the biocomposites with the largest wood dust amount. The results of the presented study are also in line with Morreale et al. They studied the wood flour influence on biopolymers and observed a similar increase in modulus values and a decrease in strain with increasing wood flour addition and a slight change in the tensile strength of the examined samples [35].

## 4. Conclusions

In this work, five different contents of wood dust were added to starch-based biopolymers. The obtained biocomposite films were tested in terms of mechanical, surface, and barrier properties. An increase in wood dust content caused the Young’s modulus values to rise with a progressive decrease in the max. strain. Therefore, the biocomposite films obtained became stiffer and less prone to deformation as the wood dust content increased, whereas the wood dust addition did not affect the wetting properties. All of the films, regardless of the amount of additive, had a hydrophilic surface.

Wood dust filler for polymer matrices had its properties modified, improving stiffness and immunity to dissolution. The resultant composite films facilitated the efficient management of waste materials stemming from wood processing and facilitated a concomitant reduction in the consumption of raw biopolymer resources. Furthermore, this bio-sourced material holds promise for applications within the packaging industry and agriculture sectors, thus substantively contributing to mitigating the ecological footprint associated with conventional plastic materials. This cheaper alternative to conventional polymers can be used in bags, films, mulching, and covering foils, which easily decompose in the environment.

## Figures and Tables

**Table 1:** The values of the hemicellulose, cellulose, and lignin contents. The fibre components were determined via the Van Soest method using an ANKOM 2000 Fiber Analyzer.

	NDF	ADF	Hemi-Cellulose	Acid Lignin	Cellulose	Lignin Weight	Lignin	Σ H C L	Σ_med_ H C L	Total Amount
	%	%	%	%	%	g	%	%	87.123	%
1	82.580	75.245	7.335	27.582	47.663	0.151	30.558	85.556		
2	85.356	77.870	7.486	35.294	42.576	0.203	39.725	89.788		95.93
3	85.905	78.729	7.176	32.390	46.339	0.156	32.509	86.024		

**Table 2:** The values of the roughness and contact angle; data are given are mean ± standard deviation (SD).

Film	S_a_ (nm)	S_q_ (nm)	Contact Angle (°)
W0	229 ± 18	282 ± 31	38.1 ± 1.4
W5	108 ± 1	132 ± 2	32.8 ± 3.6
W10	150 ± 6	200 ± 2	33.0 ± 7.1
W15	101 ± 16	127 ± 14	57.5 ± 4.5
W20	116 ± 35	154 ± 28	37.2 ± 1.9
W25	99 ± 17	127 ± 21	20.3 ± 2.8

**Table 3:** CIE L*a*b, Chroma, and Hue parameters; data given are mean ± standard deviation (SD).

	B_G	W0	W5	W10	W15	W20	W25
L*	97.133 ± 0	93.757 ± 0.107	92.332 ± 0	90.234 ± 0.333	89.645 ± 0.660	87.144 ± 1.191	89.958 ± 0.695
a*	−0.699 ± 0	−0.823 ± 0.259	−0.877 ± 0	−1.118 ± 0.259	−1.023 ± 0.164	−0.823 ± 0.146	−1.037 ± 0.091
b*	1.918 ± 0	2.616 ± 0.251	6.789 ± 0	9.472 ± 0.487	13.704 ± 1.382	16.229 ± 1.723	12.054 ± 0.266
H	−1.22 ± 0	−1.269 ± 0.08	−1.442 ± 0	−1.454 ± 0.025	−1.496 ± 0.015	−1.519 ± 0.014	−1.485 ± 0.006
C	0.0 ± 0	0.736 ± 0.270	4.874 ± 0	7.568 ± 0.491	11.791 ± 1.382	14.312 ± 1.721	10.142 ± 0.269

**Table 4:** The mechanical parameters of the films; data given are mean ± standard deviation (SD).

	W0	W5	W10	W15	W20	W25
Thickness (mm)	0.117 ± 0.02	0.167 ± 0.02	0.179 ± 0.01	0.207 ± 0.04	0.217 ± 0.01	0.228 ± 0.01
Tensile Strength σ_max_ (MPa)	2.38 ± 0.33	2.67 ± 0.30	2.61 ± 0.28	2.73 ± 0.22	2.78 ± 0.17	2.56 ± 0.16
Young’s Modulus E (MPa)	24.92 ± 4.31	45.27 ± 8.18	42.81 ± 6.35	46.75 ± 5.50	52.36 ± 5.95	38.80 ± 3.06
Maximum Strain ε_max_ (–)	0.42 ± 0.15	0.24 ± 0.03	0.23 ± 0.03	0.19 ± 0.02	0.18 ± 0.02	0.19 ± 0.03

## Data Availability

The data that support the findings of this study are available from the corresponding authors upon reasonable request.
